# Widespread Verrucous Plaques of Chromoblastomycosis: A 12‐Year Diagnostic Odyssey

**DOI:** 10.1002/ccr3.71147

**Published:** 2025-10-05

**Authors:** Elisha Shrestha, Anupa Sharma, Manisha Gartaula, Aniket Basnet

**Affiliations:** ^1^ Dhulikhel Hospital Kathmandu University Hospital Dhulikhel Nepal; ^2^ Tribhuvan University Teaching Hospital Kathmandu Nepal

**Keywords:** chromoblastomycosis, deep fungal infection, fungi, verruca

## Abstract

Chromoblastomycosis is a deep fungal infection of the skin and subcutaneous tissue. This case underscores the critical importance of thorough laboratory investigations, particularly histopathology, in chronic dermatological conditions that mimic other granulomatous diseases. Misdiagnosis, such as treating chromoblastomycosis as cutaneous tuberculosis without confirmatory testing, can delay appropriate therapy and prolong morbidity.

## 1 Introduction

Chromoblastomycosis (CBM) is a chronic fungal infection of the skin and subcutaneous tissue caused by a group of several species of dematiaceous (darkly pigmented) fungi—Fonsecaea pedrosoi, Phialophora verrucosa, Cladophialophora carrionii, Wangiella dermatitidis, Exophiala spinifera, and Cladophialophora boppii [1].

The etiologic agents enter the skin through traumatic implantation of contaminated material mostly on the extremities of outdoor workers [2].

It develops slowly and initially may resemble a dermatophyte infection or begin as a papule then subsequently develop into plaques, nodules, or verrucous and exophytic lesions. It may develop into tumoral, cauliflower‐like masses or may extend centripetally, leaving central areas of scarring and healing, leaving sclerotic plaques or keloids [3]. Severe and extensive forms with involvement of cutaneous, subcutaneous regions are due to lymphatic, hematogenous, or autoinoculation, which are uncommon and difficult to treat [3, 4].

Nowadays, CBM is characterized as a Neglected Tropical Disease (NTD) because it affects poor populations in tropical and subtropical areas, causing high morbidity, social stigma, and discrimination [5].

This case reports a cutaneous form of extensive Chromoblastomycosis initially misdiagnosed as tuberculosis verrucosa cutis and given antitubercular therapy twice in the past without any improvement of symptoms.

## 2 Case History and Examination

A 56‐year‐old female presented to our dermatology clinic with a chief complaint of multiple elevated warty lesions on her face, neck, and chest, as well as on bilateral upper and lower limbs for 12 years. It started as an elevated skin‐colored soft lesion on the right thigh with mild itching, which changed into a reddish fleshy lesion over 2–3 months, then developed slowly into a warty growth over a year. It gradually increased in size in a linear fashion and progressed to involve the whole of the lower and upper extremities, with a few on her chest and back. Initially, there were a few discrete warty lesions that gradually increased in number, which later coalesced and appeared larger, forming a linear branched pattern. The warty surface fell off, leaving behind hypertrophic to atrophic erythematous plaques over a few years.

She was diagnosed as tuberculosis verrucosa cutis 11 years back in other center without proper documentation and was given anti tuberuculosis treatment (ATT) for 6 months. The lesions slightly decreased in size but did not resolve. She was again started on ATT for 6 months 9 years back without complete resolution. Since then she has been on and off unknown topical medications without improvement. Over last 1 year, similar warty lesions appeared to involve face, breast, and bilateral hands.

No history of trauma or inoculation that was recalled. There was no history of weight loss, chronic cough, and lymphadenopathy.

**FIGURE 1:**
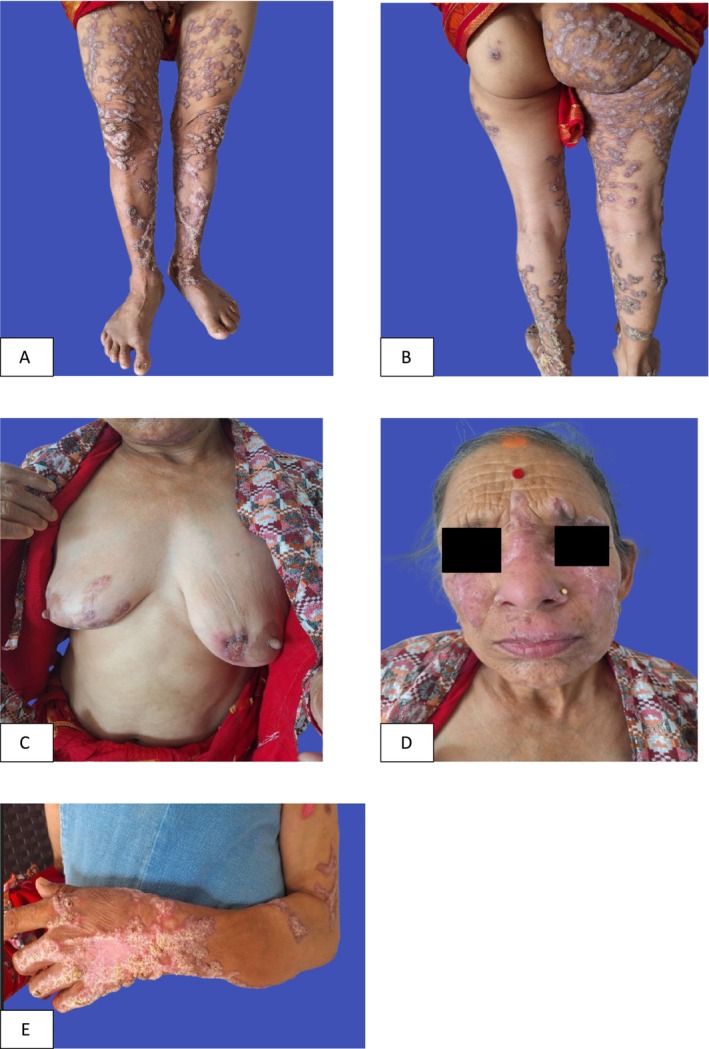
(A–E) Multiple well demarcated erythematous hyperkeratotic plaques in reticular pattern over different parts of the body.

Physical examination revealed multiple, well‐demarcated, violaceous, discrete to confluent hyperkeratotic, verrucous plaques coalescing with one another present symmetrically bilaterally over the extensor of lower extremities extending from thighs down to the feet, and more over the buttocks, flexural aspect of the right thigh and legs. Similar lesions were present on the right dorsum of the hands, face, and left breast with interspersed areas of hypertrophic and atrophic shiny erythematous plaques. A single, well‐defined, irregular shallow ulcer with yellowish exudate on the floor and crusts on the peripheral margin surrounded by erythema present on the lower inner quadrant of the right breast (Figure 1A–E).

## 3 Methods

Given the chronic and progressive nature of the patient's lesions, several differential diagnoses were considered, including cutaneous tuberculosis, chromoblastomycosis, verrucous sarcoidosis, lupus vulgaris, verrucous carcinoma, and generalized verrucous discoid lupus erythematosus (DLE). A systematic diagnostic algorithm—incorporating clinical evaluation, dermoscopy, histopathology, microbiological tests, and relevant immunological markers—is crucial for narrowing down the diagnosis in such presentations.

Her routine hematological and biochemical parameters were within normal limits, chest Xray, Matoux test revealed no gross abnormalities.

**FIGURE 2:**
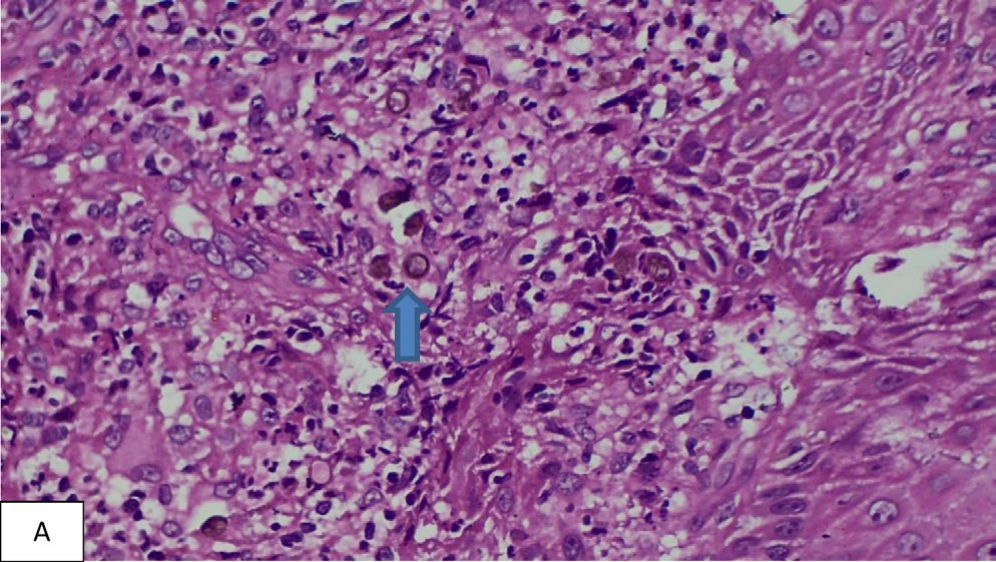
Histological section (Hematoxylin and eosin stain 400×) of the skin lesion showed neurtophilic infiltrates with presence of thick walled, dark‐brown fungal cells called sclerotic bodies, also known as Medlar bodies or copper penny bodies (Blue arrow).

Histopathology examination (HPE) was crucial revealing pseudo‐epitheliomatous hyperplasia, acanthosis, dermis showed numerous langhans type giant cells, epitheliod histiocytes, lymphocytes and occasional round dark brown thick‐walled sclerotic bodies (Medlar bodies) shown in Figure 2 confirming the diagnosis of chromoblastomycosis.

**FIGURE 3:**
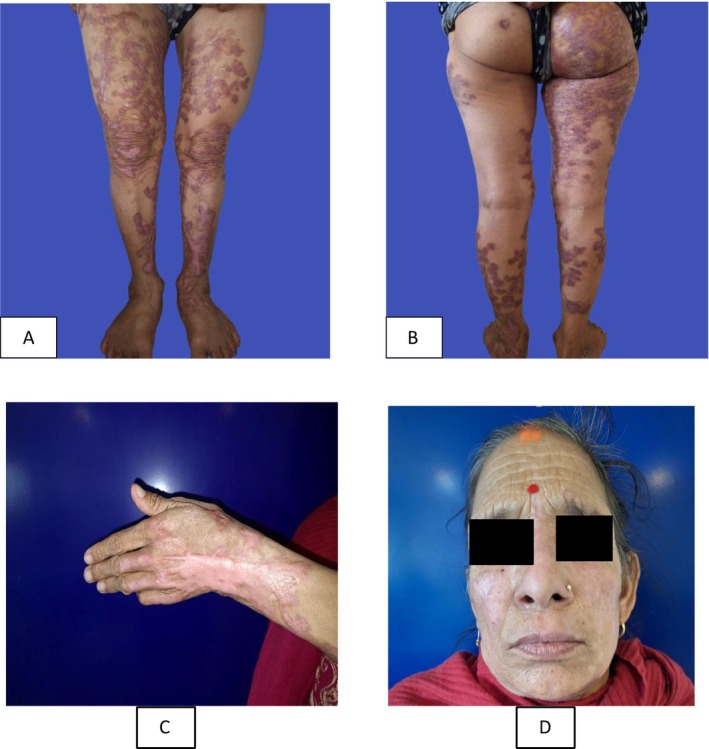
(A–D) Clinical pictures of the patient after 2 weeks of treatment with oral itraconazole.

Patient was initially started on oral itraconazole 400 mg per day along with topical antifungal (Cream Clotrimazole) application for 2 weeks. By the end of the 2nd week, most of the verrucous plaque fell off, so it was continued for another 1 month (Figure 3A–D).

## 4 Conclusion and Results

This case highlights the diagnostic challenges of chromoblastomycosis, particularly when the condition mimics other chronic dermatological disorders and is compounded by delayed or inappropriate clinical investigation. The 12‐year misdiagnosis underscores the importance of maintaining a high index of suspicion for rare fungal infections, especially in patients with nonhealing cutaneous lesions resistant to conventional therapies. Histopathological evaluation and fungal culture remain pivotal in achieving an accurate diagnosis. This case serves as a reminder for clinicians to consider chromoblastomycosis in differential diagnoses and to adopt a multidisciplinary approach when managing atypical or recalcitrant dermatological conditions.

## 5 Discussion

Chromoblastomycosis is a chronic granulomatous infection of the skin and subcutaneous tissue caused by several dematiaceous fungi most commonly *Cladophialophora carrionii* and *Fonsecaea pedrosoi*, which are present in soil, woods, and plant debris and get inoculated after penetrating cutaneous injury [6]. As an agricultural worker, our patient might have suffered a similar minor injury that she couldn't seem to remember.

Chromoblastomycosis is an indolent cutaneous infection, which can present as nodular, plaque‐like, verrucous, or cicatrical lesions [3]. In a study done by Pradhan SV et al. in 2007, 13 cases of chromoblastomycosis were diagnosed during the period of 1999–2006 in Nepal who presented as nodular or verrucous growths [7].

Clinically, the condition may simulate tuberculosis verrucosa cutis, squamous cell carcinoma, and sporotrichosis [6]. Initially, this case was also misdiagnosed as tuberculosis verrucosa cutis clinically and underwent treatment for the same, with no response. Pradeepkumar et al. [8] and Bandyopadhyay et al. [9] also reported cases of chromoblastomycosis mimicking cutaneous tuberculosis.

Histological examination is a useful tool to establish diagnosis. Hyperkeratosis, pseudo‐epitheliomatous hyperplasia, mixed‐tissue inflammatory response with acute and chronic inflammation, and granulomas with giant cells, medlar bodies, and the thick‐walled pigmented copper bodies in the dermis are consistent features [10]. Microscopic examination with 10% KOH examination of skin scraping, and fungal cultures can also be done for its diagnosis [11].

Treatment of chromoblastomycosis is difficult. Different treatment modalities include topical and systemic antifungal agents, conventional surgery, thermotherapy, cryotherapy, LASERS, photodynamic therapy, or a combination of any of these [12]. In this patient, after starting oral itraconazole, her lesions improved as some of the verrucous plaques began to thin and fall off within 2 weeks of initiating oral itraconazole therapy.

## Author Contributions

**Elisha Shrestha:** conceptualization, formal analysis, investigation, methodology, supervision, visualization, writing – original draft, writing – review and editing. **Anupa Sharma:** data curation, formal analysis, investigation, writing – original draft, writing – review and editing. **Manisha Gartaula:** investigation, resources. **Aniket Basnet:** investigation, writing – review and editing.

## Ethics Statement

The authors have nothing to report.

## Consent

Written informed consent was obtained from the patient for the publication of this report following the journal's patient consent policy.

## Conflicts of Interest

The authors declare no conflicts of interest.

## Data Availability

The authors of this manuscript are prepared to provide Supporting information concerning the case report upon official request. All data generated or analyzed during this study are included in this article. Further inquiries can be directed to the corresponding authors.

## References

[1] O. Lupi , S. K. Tyring , and M. R. McGinnis , “Tropical Dermatology: Fungal Tropical Diseases,” Journal of the American Academy of Dermatology 53, no. 6 (2005): 931–951. 16310053 10.1016/j.jaad.2004.10.883

[2] M. J. Najafzadeh , A. Rezusta , M. I. Cameo , et al., “Successful Treatment of Chromoblastomycosis of 36 Years Duration Caused by Fonsecaea Monophora,” Medical Mycology 1–4 (2009): 1–4. 10.1080/13693780903008813 19488919

[3] M. Ameen , “Chromoblastomycosis: Clinical Presentation and Management,” Clinical and Experimental Dermatology 34, no. 8 (2009): 849–854. 19575735 10.1111/j.1365-2230.2009.03415.x

[4] G. K. Verma , S. Verma , G. Singh , et al., “A Case of Extensive Chromoblastomycosis From North India,” Brazilian Journal of Microbiology 45, no. 1 (2014): 275–278. 24948945 10.1590/S1517-83822014005000025 PMC4059311

[5] D. W. C. L. Santos , C. D. M. P. E. S. De Azevedo , V. A. Vicente , et al., “The Global Burden of Chromoblastomycosis,” PLoS Neglected Tropical Diseases 15, no. 8 (2021): e0009611. 34383752 10.1371/journal.pntd.0009611 PMC8360387

[6] G. Kurien , K. Sugumar , N. C. Sathe , and V. Chandran , Chromoblastomycosis (StatPearls Publishing, 2024), http://www.ncbi.nlm.nih.gov/books/NBK470253/. 29261968

[7] S. Pradhan , O. Talwar , A. Ghosh , R. Swami , K. Shiva Raj , and S. Gupta , “Chromoblastomycosis in Nepal: A Study of 13 Cases,” Indian Journal of Dermatology, Venereology and Leprology 73, no. 3 (2007): 176–178. 17558050 10.4103/0378-6323.32741

[8] N. S. Pradeepkumar and N. M. Joseph , “Chromoblastomycosis Caused by Cladophialophora Carrionii in a Child From India,” Journal of Infection in Developing Countries 5, no. 7 (2011): 556–560. 21795827 10.3855/jidc.1392

[9] A. Bandyopadhyay , K. Majumdar , M. Gangopadhyay , and S. Banerjee , “Cutaneous Chromoblastomycosis Mimicking Tuberculosis Verrucosa Cutis: Look for Copper Pennies!,” Turkish Journal of Pathology 31 (2013): 223–225. 10.5146/tjpath.2013.01197 24272932

[10] M. Shenoy , B. Girisha , and S. Krishna , “Chromoblastomycosis: A Case Series and Literature Review,” Indian Dermatology Online Journal 14, no. 5 (2023): 665–669. 37727562 10.4103/idoj.idoj_292_23 PMC10506812

[11] N. Damayanti , L. Noor , H. Purwanto , and A. Siswati , “Diagnosis and Therapy of Chromoblastomycosis,” Journal of Pakistan Association of Dermatologists 32, no. 2 (2022): 443–448.

[12] D. K. Khadka , D. Pandey , and S. Agrawal , “Combination Treatment for Extensive Chromoblastomycosis: A Case Report,” Nepal Journal of Dermatology, Venereology & Leprology 19, no. 2 (2021): 58–61.

